# Large Pancreatic Gastrointestinal Stromal Tumor: A Diagnostic Quandary

**DOI:** 10.7759/cureus.103297

**Published:** 2026-02-09

**Authors:** Pranav S Ramamurthy, Syed H Sohail, Leo Montesinos-Barona, Nha T Duong

**Affiliations:** 1 Internal Medicine, UMass Chan Medical School - Baystate Medical Center, Springfield, USA; 2 Gastroenterology, UMass Chan Medical School - Baystate Medical Center, Springfield, USA; 3 Pathology, UMass Chan Medical School - Baystate Medical Center, Springfield, USA

**Keywords:** conservative management of gist, extragastrointestinal stromal tumors, incidental tumor, large gastrointestinal stromal tumor, pancreatic gist

## Abstract

Gastrointestinal stromal tumors (GISTs) are rare mesenchymal neoplasms typically arising from the gastrointestinal tract, most often in the stomach or small intestine. Primary pancreatic GIST is exceedingly rare, with fewer than 50 cases reported in the literature. We present a unique case of a 77-year-old female who was found to have a 6.5 cm complex mass in the left abdomen with inconclusive imaging findings. Endoscopic ultrasound (EUS) further characterized the lesion as a mixed solid and cystic mass with peripheral enhancement, but its origin remained uncertain. Pathology revealed a spindle cell type GIST. Given the diagnostic ambiguity, the patient underwent diagnostic laparoscopy, which definitively identified the mass as originating from the pancreatic tail, confirming the rare diagnosis of primary pancreatic GIST. Despite recommendations for surgical resection or treatment with imatinib, the patient declined both and chose to pursue close surveillance. The patient's clinical condition remained stable one year after initial diagnosis. This case underscores the importance of including pancreatic GIST in the differential diagnosis of atypical pancreatic masses, especially those with mixed solid and cystic components. It also highlights the role of EUS as well as laparoscopy in resolving diagnostic uncertainty for complex abdominal masses. Further studies are needed to better understand the natural history and optimal management of low-risk, asymptomatic pancreatic GISTs. This case also highlights the importance of shared decision-making in the management of conditions where guidelines are not well-developed.

## Introduction

Gastrointestinal stromal tumors (GISTs) are rare mesenchymal neoplasms originating from or differentiating toward interstitial cells of Cajal (ICCs), the pacemaker cells of the gastrointestinal (GI) tract. They typically exhibit spindle cell, epithelioid, or pleomorphic morphology and are immunopositive for KIT (CD117) and DOG1, with KIT or PDGFRA mutations present in about 85% of cases [1]. While GISTs most commonly arise in the stomach or small intestine, rare extracellular GISTs, including pancreatic GISTs, have been reported [2].

These tumors likely develop from KIT-positive ICC-like cells within the pancreas, which regulate ductal and vascular motility. The discovery of c-KIT-positive ICCs in the pancreas suggests a possible origin for pancreatic GISTs, an extremely rare but documented entity. These pancreatic ICC-like cells, located around the main duct and large vessels, may undergo oncogenic transformation through mutations in KIT or PDGFRA, mirroring the pathogenesis of GI tract GISTs. Their presence supports the idea that GISTs can arise in any location where ICCs exist, extending beyond the traditional GI tract. Pancreatic GISTs are exceedingly rare, with only about 50 cases reported in the literature.

Given the rarity and diagnostic complexity of pancreatic GISTs, we present a case in which multiple cross-sectional imaging modalities and endoscopic imaging were unable to provide a conclusive diagnosis despite biopsy-proven GIST, which eventually necessitated a laparoscopy to confirm the diagnosis of a large pancreatic GIST, a scenario that has not been reported prior. Furthermore, our patient decided to proceed with clinical monitoring rather than active therapy, which highlights the importance of shared decision-making in these rare clinical scenarios.

## Case presentation

**Figure 1:**
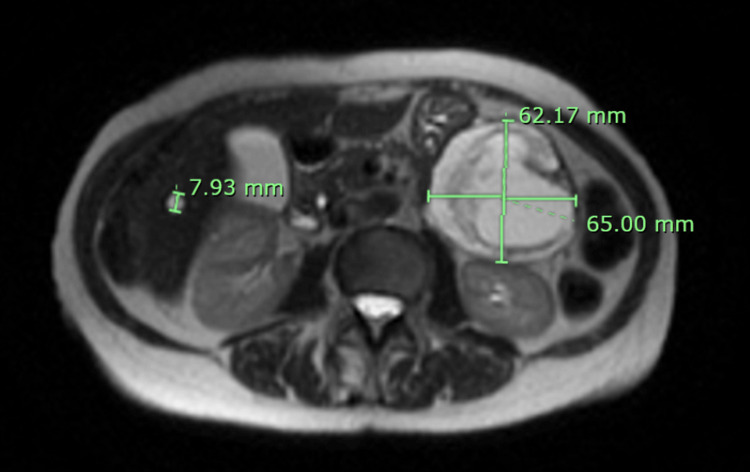
MRI appearance: axial SS-FSE sequence MRI of the abdomen without contrast (T2-weighted SS-FSE) demonstrating a complex mixed solid and cystic mass with peripheral enhancement and restricted diffusion, possibly arising from the pancreatic tail, which was suspicious for malignancy with no evidence of metastatic disease. SS-FSE, single-shot fast spin-echo; MRI, magnetic resonance imaging

**Figure 2:**
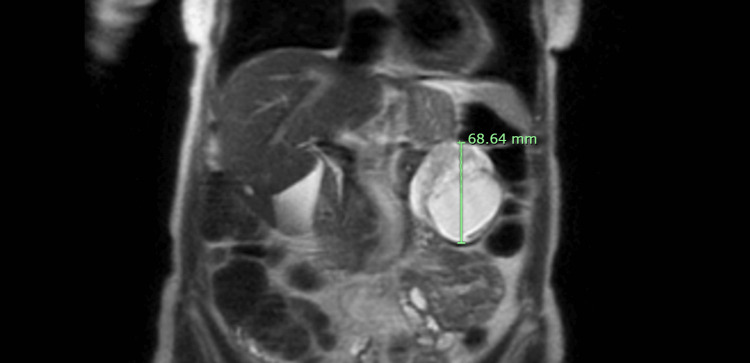
MRI appearance: coronal SS-FSE sequence MRI of the abdomen without contrast (T2-weighted SS-FSE) demonstrating an alternate view of a complex mixed solid and cystic mass with peripheral enhancement and restricted diffusion, possibly arising from the anterior aspect of the pancreatic tail but also apparently continuous with the posterior stomach. SS-FSE, single-shot fast spin-echo; MRI, magnetic resonance imaging

A 77-year-old female with a past medical history of left-sided carotid stenosis without any significant surgical history and no home medications presented with an isolated episode of nonspecific abdominal pain. She denied any other associated symptoms, such as nausea, vomiting, or weight loss, and did not report any family history of malignancy. Of note, the patient had a history of declining all vaccinations due to concerns about side effects. Her vitals were unremarkable. Her physical examination revealed no abdominal tenderness or concerning findings and was unremarkable. Her blood work was unremarkable apart from a mild elevation in alkaline phosphatase to 145 IU/L. The patient underwent a computed tomography (CT) scan of her abdomen and pelvis, which revealed an approximately 62x62x71 mm mass in the left abdomen with an unclear site of origin. After her initial episode, the patient’s abdominal pain resolved and did not recur. The patient then underwent further imaging with magnetic resonance imaging (MRI) of her abdomen, showing a 65.00x62.17x68.64 mm complex mixed solid and cystic mass with peripheral enhancement and restricted diffusion (Figure 1 and Figure 2). The mass was noted to possibly arise from the pancreatic tail, which was suspicious for malignancy with no evidence of metastatic disease.

**Figure 3:**
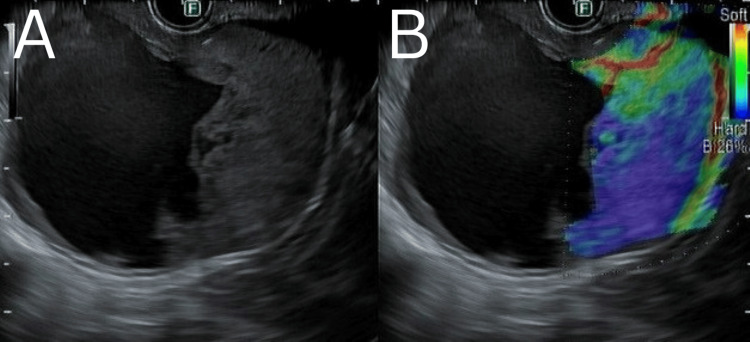
EUS with elastography EUS imaging (A) demonstrates a mixed solid and cystic lesion, with elastography (B) demonstrating an increase in stiffness of the solid component consistent with a mass. Both the cystic component and the solid component were sampled. EUS, endoscopic ultrasound

The patient underwent an endoscopic ultrasound (EUS), which showed a 60x60 mm heterogeneous lesion arising from the region at the tail of the pancreas, although it was not definitively localizable (Figure 3). The lesion had an anechoic center consistent with cystic fluid with a few hyperechoic densities suspected to be sludge or debris. The periphery of the lesion was hypoechoic, concerning for a solid mass. The cystic area of the lesion was aspirated, which yielded about 20 cc of thin maroon fluid. The solid component of the lesion was sampled with a fine-needle biopsy as well. No malignant lymph nodes were noted in the periportal, pancreatic, or celiac area. The tail of the pancreas contained hyperechoic strands and a normal caliber pancreatic duct. The gallbladder contained multiple small hyperechoic densities consistent with stones as well as sludge. The common bile duct was normal in caliber without any filling defects. The head of the pancreas also appeared normal without any lesions. Given the difficulty localizing the origin of the mass, the differentials at this time included pancreatic malignancies such as a pancreatic mucinous neoplasm or a potential GIST arising from the posterior stomach or pancreas.

**Figure 4:**
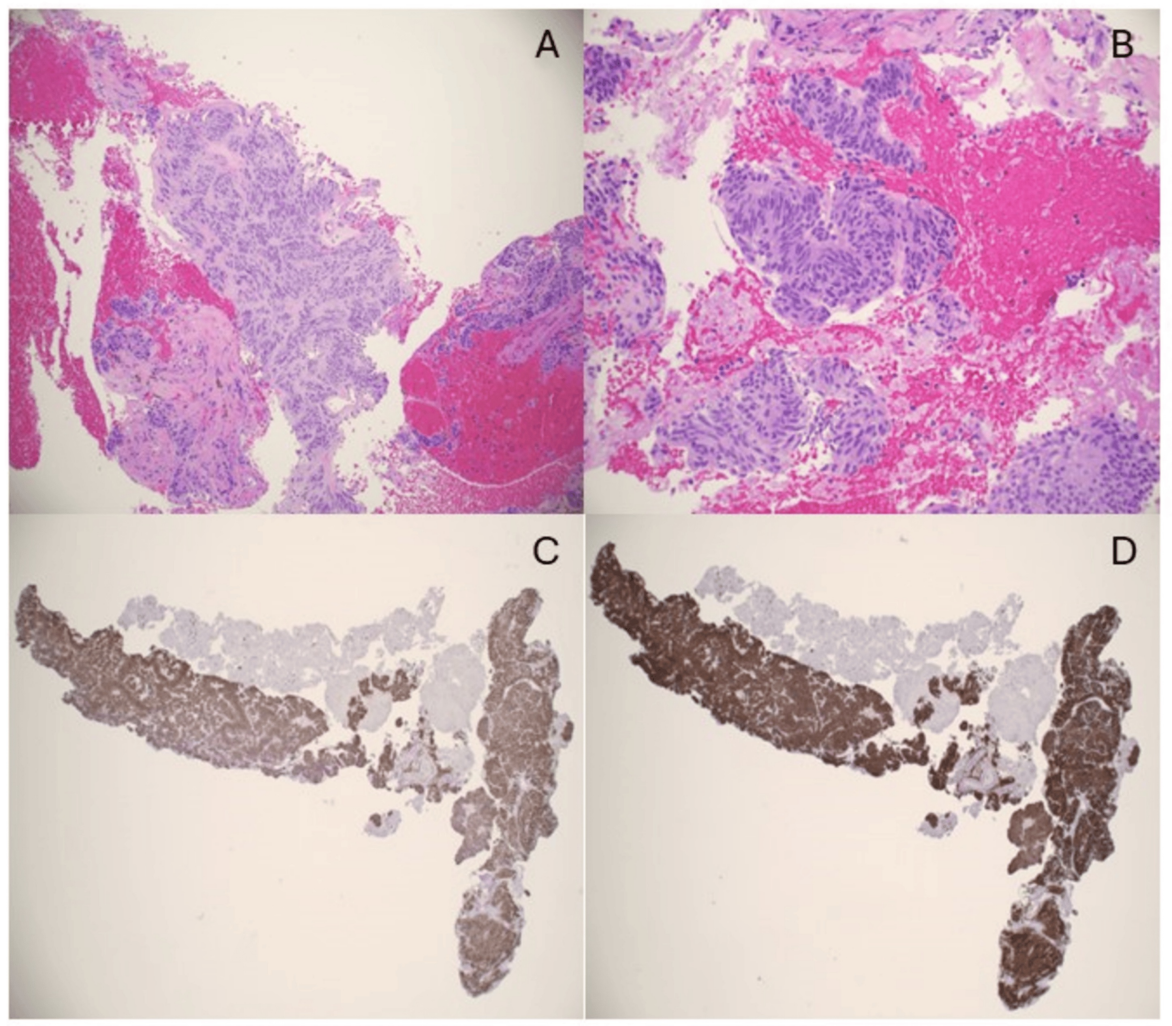
Pathology Pathology slides showing (A) pancreatic GIST, middle power; (B) pancreatic GIST, high power; (C) C-KIT positive stain; and (D) DOG-1 positive stain. GIST, gastrointestinal stromal tumors

The pathology report revealed a diagnosis of a GIST with spindle cell type (Figure 4). The mitotic rate was zero with a histologic grade of one. Molecular analysis was also performed using the two-site tumor 15 NGS panel, which revealed a KIT V560D mutation. The patient was referred to the surgical and medical oncology. Based on the information available, this was thought to be a GIST, and treatment with imatinib was discussed [3,4]. The patient was generally opposed to taking any medications and, after discussion, opted for a diagnostic laparoscopy for definitive diagnosis and localization, though she opposed surgical resection during the same procedure.

Diagnostic laparoscopy showed omental atrophy without obvious peritoneal implants or lesions. The stomach was inspected but was without obvious pathology. The large mass was noted to be separate from the posterior stomach wall and originated from the tail of the pancreas. This confirmed the diagnosis of pancreatic GIST.

The patient was offered both surgical resection and imatinib; however, she opted against both medical and surgical treatment and elected to undergo serial monitoring with oncology. She had significant concerns regarding taking imatinib or undergoing further surgery, and so, considering her low-grade pathology, it was decided that monitoring would be a reasonable option pending further progression of the disease. Her blood work has remained within normal limits for about 12 months. Repeat imaging every six months was initially suggested; however, the patient was initially hesitant to pursue repeat imaging due to difficulty undergoing MRI. She also continued to decline imatinib despite numerous discussions with oncology. Currently, the patient continues to report no symptoms from her disease over a year after the diagnosis, and thus, monitoring is being continued pending repeat abdominal MRI following further shared decision-making with the patient.

## Discussion

Pancreatic GISTs represent an exceptionally rare subset of extragastrointestinal stromal tumors (EGISTs), with fewer than 50 cases reported in the literature to date. The management strategies for EGISTs are largely extrapolated from gastric and small bowel GISTs due to limited data [1]. Due to their rarity, pancreatic GISTs are poorly characterized, and much of the available knowledge is derived from isolated case reports and small retrospective series. Consequently, their natural history, optimal diagnostic approach, and management strategies remain incompletely defined [5,6].

Pancreatic GISTs are thought to arise from KIT-positive ICC-like cells identified within the pancreas, particularly around ducts and vascular structures. These cells may undergo oncogenic transformation via activating KIT or PDGFRA mutations, mirroring the pathogenesis of conventional GISTs [3,5]. Clinically, pancreatic GISTs often present with nonspecific or minimal symptoms, including vague abdominal pain or incidental detection on imaging [7,8]. Based on prior reviews, they occur more commonly in females with a median age of diagnosis in the 50s [8,9]. Our patient’s isolated abdominal pain and prolonged asymptomatic course align with prior observations of low-risk GISTs demonstrating relatively indolent behavior [2,6].

Most reported pancreatic GISTs occur in the pancreatic head and frequently present as large masses at the time of diagnosis, often exceeding 5 cm in size [2,7]. Similar to prior reports, the tumor in our patient was sizable at presentation (>6cm), though it was located in the pancreatic tail, which is a less common location. However, in contrast to most published cases, our patient’s tumor demonstrated a mitotic index of zero and low histologic grade. In the largest clinicopathologic review of 44 patients with pancreatic GISTs, Liu et al. demonstrated that pancreatic GISTs have significantly worse disease-free survival (DFS) compared with gastric GISTs, despite similar molecular profiles, with over 85% of pancreatic GISTs being classified as high risk [2]. Importantly, mitotic index was the only factor independently associated with DFS, underscoring the prognostic importance of tumor biology over size or location alone. Another review of nine patients by Gupta et al. demonstrated an association of DFS with histologic type, mitotic index, National Institutes of Health risk category, and adjuvant therapy [8].

Accurate preoperative diagnosis of pancreatic GISTs remains challenging due to their rarity and overlapping imaging features with more common pancreatic neoplasms. On cross-sectional imaging, pancreatic GISTs may appear as well-circumscribed, hypervascular masses with areas of cystic degeneration or necrosis, features that overlap with numerous other pancreatic tumors, including pancreatic neuroendocrine tumors, solid pseudopapillary neoplasms, mucinous cystic neoplasms, intraductal papillary mucinous neoplasms, and even pancreatic adenocarcinoma [10,11]. While EUS with fine-needle biopsy is essential for tissue diagnosis, it may not reliably localize tumors adjacent to the stomach and pancreas. In this case, imaging and EUS failed to establish the tumor’s origin, necessitating diagnostic laparoscopy. This approach, which is rarely described in the literature, proved critical in confirming pancreatic origin and highlights the limitations of noninvasive diagnostics in select cases [7,8].

Another distinguishing feature of this case is the extensive diagnostic evaluation required to localize the tumor origin. While prior case reports have described successful preoperative localization using CT, MRI, or EUS alone, our case demonstrates that even multimodality imaging combined with biopsy-proven GIST may be insufficient to definitively determine the site of origin. This diagnostic uncertainty had significant implications for management, as surgical planning differs substantially depending on whether a tumor arises from the stomach versus the pancreas. To our knowledge, this is the first reported pancreatic GIST in which diagnostic laparoscopy was required solely for localization and confirmation of pancreatic origin after noninvasive modalities failed to do so. This underscores a unique diagnostic challenge not well documented in existing literature.

Complete surgical resection with negative margins remains the only potentially curative treatment for localized GISTs [11,12]. For pancreatic GISTs, surgical approaches vary depending on tumor location and may include pancreaticoduodenectomy, distal pancreatectomy with or without splenectomy, or local enucleation in select cases. Several reports have demonstrated favorable outcomes following laparoscopic resection of pancreatic tail GISTs [10].

Advances in systemic therapy have significantly improved outcomes in GISTs overall. Tyrosine kinase inhibitors, particularly imatinib, remain central to treatment, with well-established efficacy in KIT- and PDGFRA-mutant tumors [3]. Emerging data suggest that immune-related mechanisms may also influence tumor behavior and therapeutic response, although their relevance in pancreatic GISTs remains poorly defined [4,9]. Molecular subtype further impacts prognosis and treatment response, even among imatinib-naive patients, reinforcing the importance of mutational analysis in rare presentations. Rossi et al. demonstrated that mutation was a significant prognostic indicator of overall survival in naive, localized GISTs, with KIT-mutated patients having a worse outcome than PDGFRA-mutated or triple-negative (KIT, PDGFRA, BRAF wild-type) cases [5]. Meta-analyses also suggest potential benefits of neoadjuvant imatinib in selected cases, particularly for large or anatomically complex tumors [7,10].

This case also underscores the importance of individualized, patient-centered decision-making in rare malignancies with limited evidence. Despite extensive counseling regarding the benefits of both surgical resection and imatinib therapy, the patient declined all active interventions, citing concerns about medication side effects, surgery, and vaccination requirements related to potential splenectomy. Given the tumor’s low mitotic index, absence of metastatic disease, and the patient’s asymptomatic status, a strategy of close surveillance was adopted through shared decision-making. While this approach deviates from standard recommendations favoring resection or immunotherapy, it reflects the realities of individualized care in rare malignancies with limited evidence and highlights the importance of respecting patient autonomy.

It remains to be seen whether serial monitoring is a reasonable approach to a very limited subset of patients with low-risk, asymptomatic pancreatic GISTs with a mitotic index of zero. Notably, as previously mentioned, pancreatic GISTs appear to have worse DFS compared to gastric GISTs, suggesting that prolonged observation without intervention may not be appropriate for most patients [7]. Therefore, individualized management incorporating tumor mitotic index, histopathologic grade, molecular analysis, anatomic considerations, patient preferences, and multidisciplinary discussion remains essential in guiding treatment decisions for pancreatic GISTs. Long-term follow-up of cases such as this one may help inform future risk stratification and management guidelines.

## Conclusions

Pancreatic GIST must be considered as a differential for any pancreatic mass, especially complex masses with both solid and cystic components. This case also highlights the role of EUS as well as laparoscopy in resolving diagnostic uncertainty for complex abdominal masses. Further studies are needed to better understand the natural history and optimal management of low-risk, asymptomatic pancreatic GISTs.
